# Supplementary material for: Neutral beam microscopy with a reciprocal space approach using magnetic beam spin encoding
Source: Nat Commun. 2024 Aug 15;15:7046. doi: 10.1038/s41467-024-51175-2 (PMC11327282; doi:10.1038/s41467-024-51175-2)
Supplement: Supplementary file 1 — Supplementary Information [file 41467_2024_51175_MOESM1_ESM.pdf]

Supplementary Information

Neutral beam microscopy with a reciprocal space approach using magnetic beam spin encoding

Morgan Lowe[1], Yosef Alkoby[1], Helen Chadwick[1], and Gil Alexandrowicz[1*]

[1] Department of Chemistry, Faculty of Science and Engineering, Swansea University, Swansea SA2 8PP, UK

Content

1. Homogeneity of the gradient within the magnetic encoding device
2. Full experimental signals for configuration A
3. Full experimental signals for configuration B
4. Schematic of magnetic encoding device
5. Simulating the effect of resolution enhancement on the SNR of images reconstructed using magnetic encoding
6. Block diagram for 1d and 2d image generation
7. Derivation of $\rho(x)$ and $\rho(x, y)$ spatial reconstructions

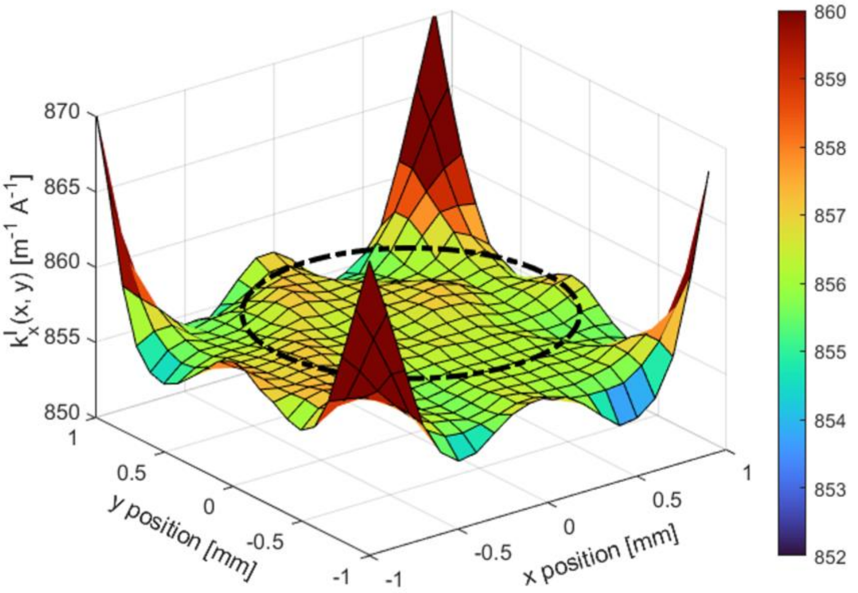

Figure S1. Homogeneity of the gradient within the magnetic encoding device. Contour plot of $k_x^I(x, y)$ calculated using magnetic field profiles generated through finite-element modelling of the $N = 12$ gradient field assembly (in ANSYS Maxwell). The black circle illustrates the $1.5mm$ diameter of the central region, corresponding to the dimensions of the ceramic tube (illustrated in figure S4) which effectively confines the maximal position of the beam particles. The flatness of the central region indicates good gradient field homogeneity.

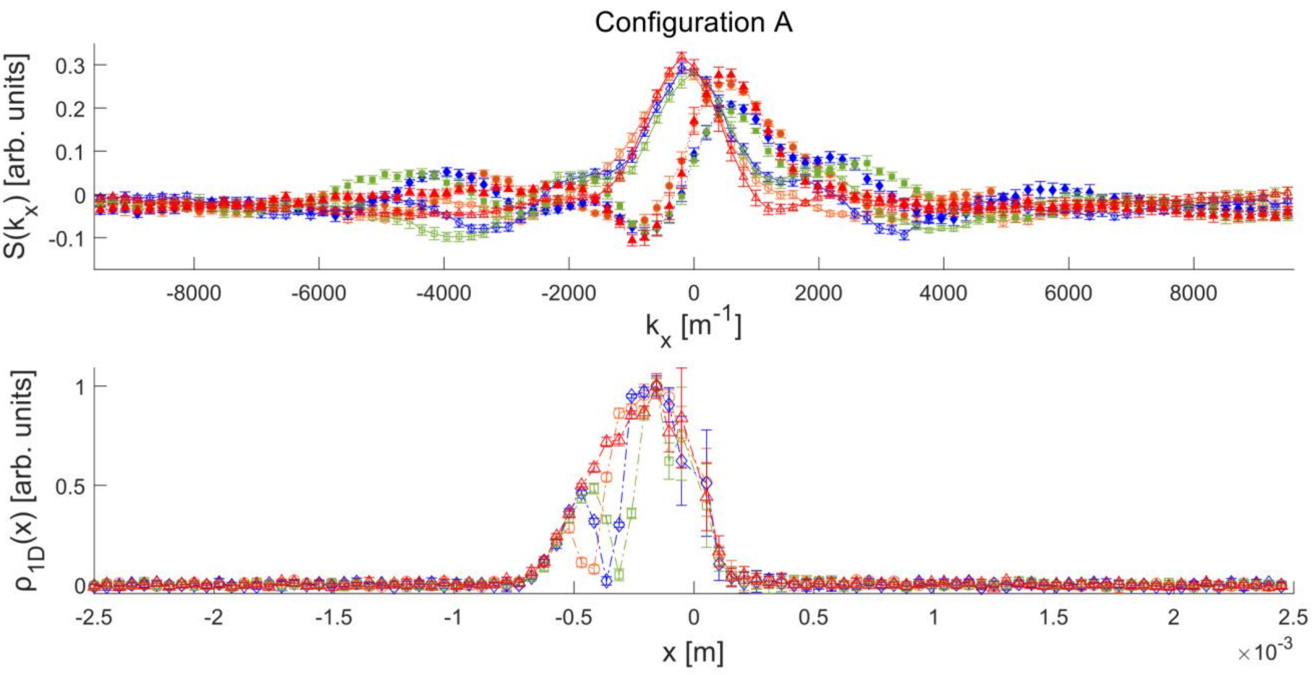

Figure S2. Full experimental signals for configuration A. All 4 $S(k_x)$ measurements (solid represents the real and dotted the imaginary components of the signal) along with the corresponding spatial profile reconstructions $\rho_{1D}(x)$. Error bars were calculated from the standard deviation of repeat measurements.

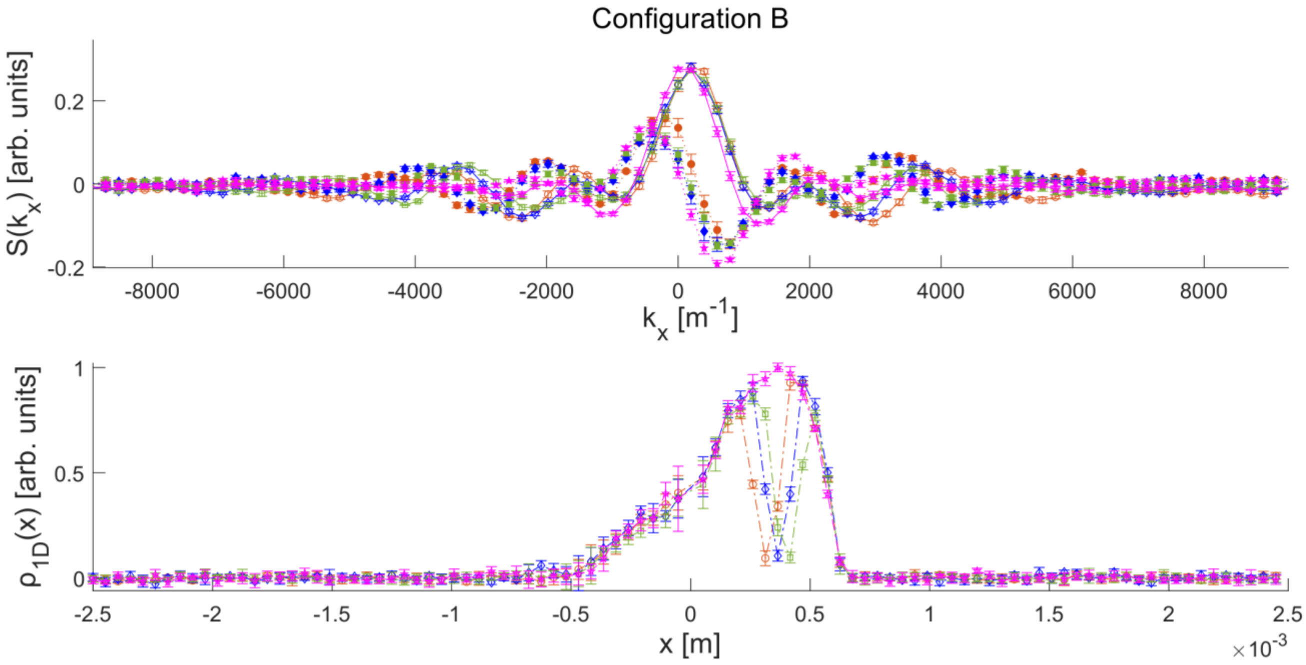

Figure S3. Full experimental signals for configuration B. All 4 $S(k_x)$ measurements (solid represents the real and dotted the imaginary components of the signal) along with the corresponding spatial profile reconstructions $\rho_{1D}(x)$. Error bars were calculated from the standard deviation of repeat measurements.

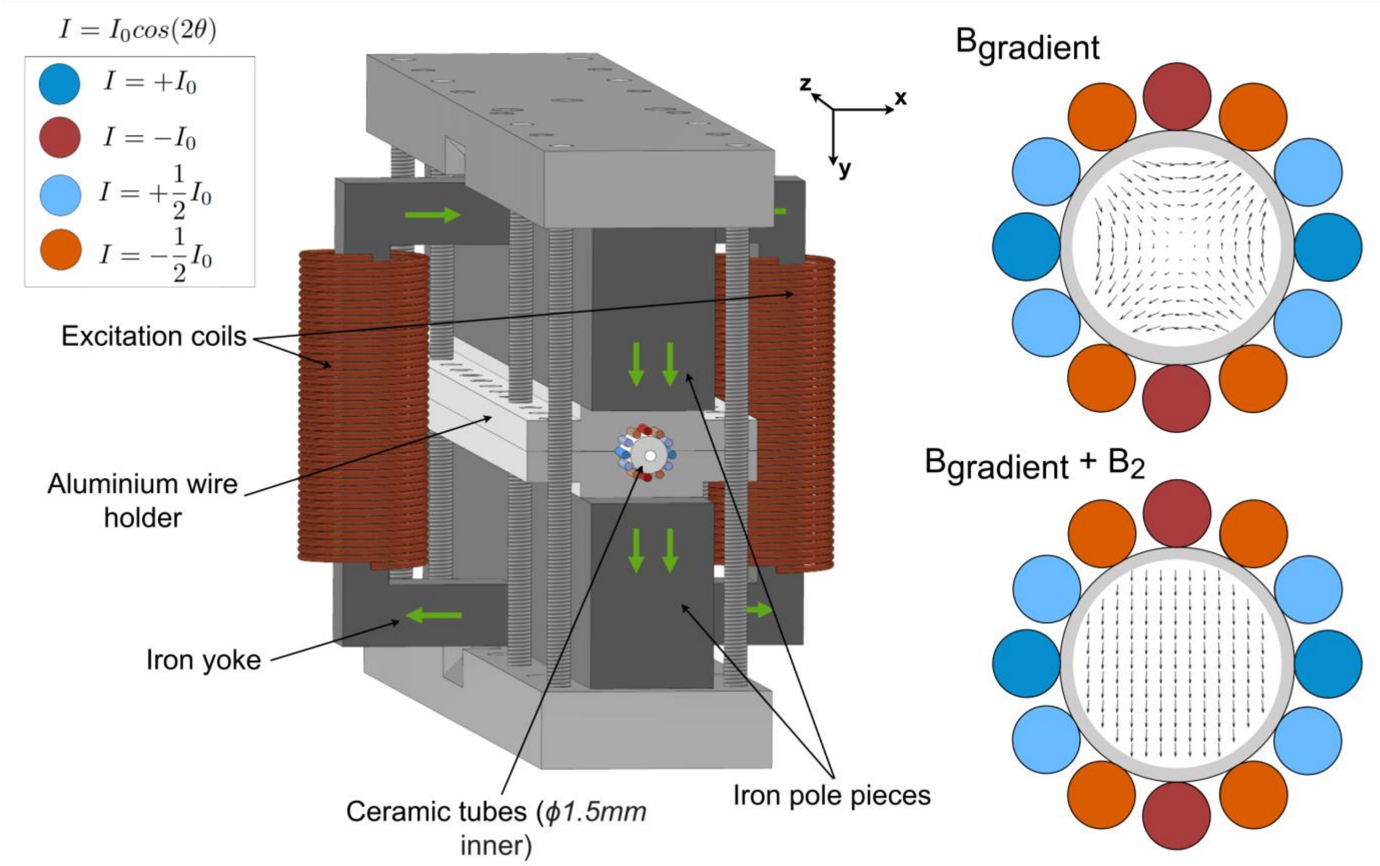

Figure S4. Schematic of magnetic encoding device. The gradient field is produced by 12 parallel wires radially distributed around a ceramic tube (the legend shows the arrangement of the wires in terms of magnitudes and current polarities). The additional homogenous field is produced by a combination of two excitation coils, driving a field through a magnetic core and between two pole pieces located above and below the gradient assembly. The Green arrows Illustrate the direction of the flux induced by the excitation coils, generating $\mathbf{B}_2$. Two vector plots are displayed to illustrate the directionality of the fields generated by both; the gradient wires, and the combination of the gradient wires and $\mathbf{B}_2$ within the confines of the $1.5mm$ diameter ceramic tube (not to scale). The inner diameter of the ceramic tube ($1.5mm$) was chosen to limit the beam passing through the encoding device to a region where the gradient is sufficiently homogeneous.

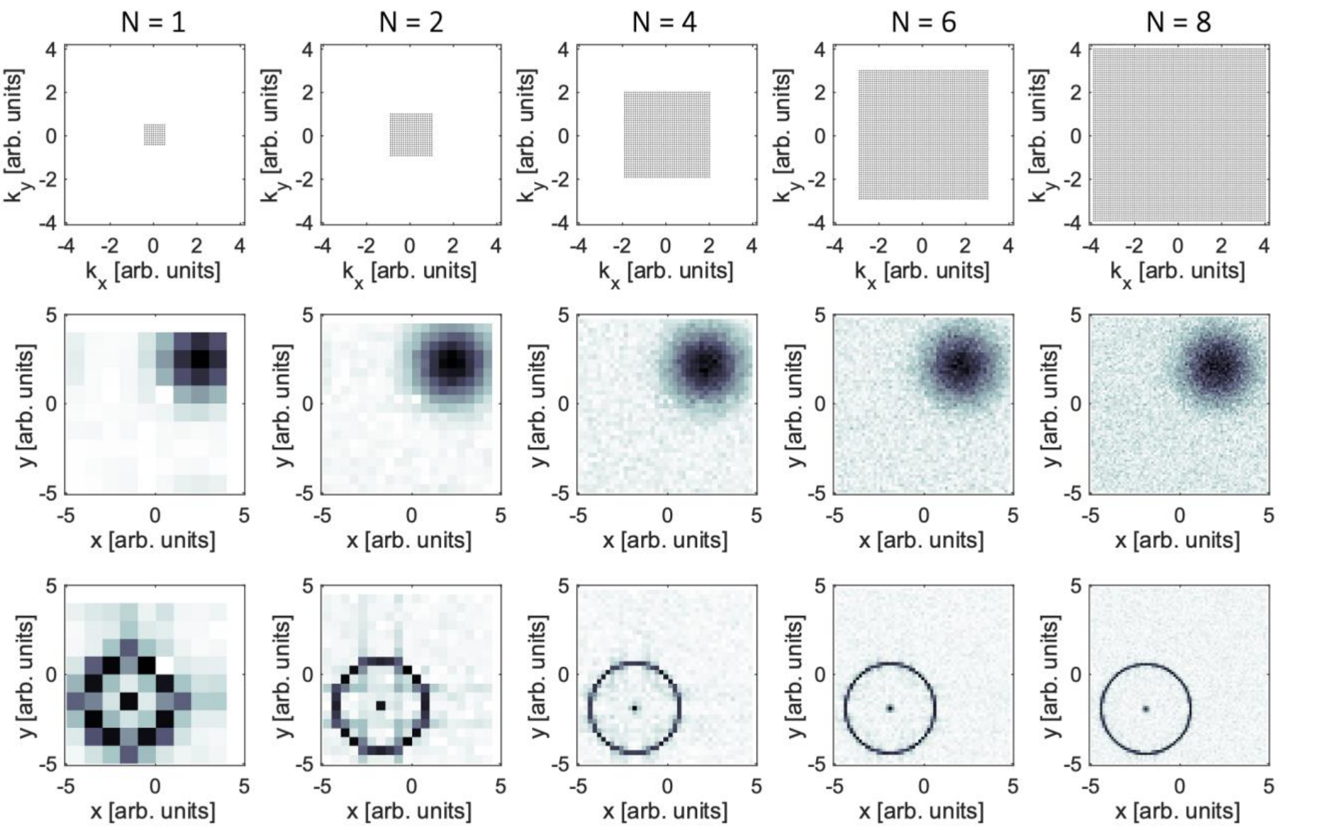

Figure S5. Simulating the effect of resolution enhancement on the SNR of images reconstructed using magnetic encoding. *The top row illustrates the* k_x, k_y *measurement points required for 5 different resolution enhancement factors (N=1,2,4,6,8) corresponding to meshes with 10x10 (left side) up to 80x80 (right side) elements. The middle and lower rows show reconstructions of* $\rho(x,y)_{smooth}$ *and* $\rho(x,y)_{sharp}$ *respectively, for these 5 different resolutions. The difference between the images shown in this figure and those shown in figure 6, in the main text, are that the measurement time for an individual* k_x, k_y *point has been kept fixed, i.e. higher resolutions have been allowed a longer total simulated measurement time.*

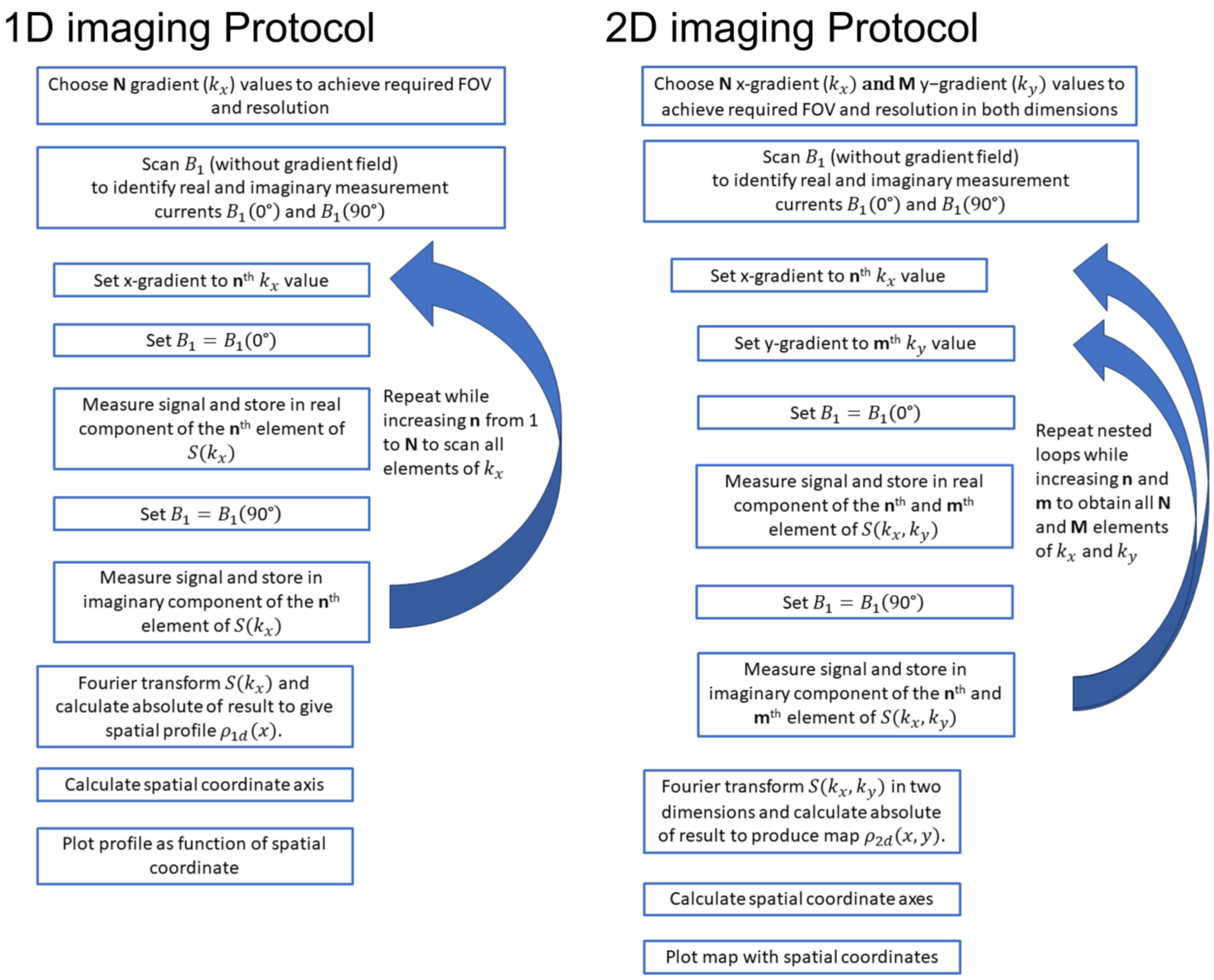

Figure S6. Block diagram for 1d and 2d image generation. Experimental protocols for magnetic encoded imaging performed in 1-dimension and 2-dimension respectively (from top to bottom).

Supplementary 7 – Derivation of $\rho(x)$ & $\rho(x,y)$ spatial reconstructions.

Here we provide a more detailed derivation of Eqs. 1 and 2 of the main text, starting from the expression for the overall precession phase at the end of the encoding field, $\phi = \gamma \frac{L}{|\bar{\mathrm{v}}|}\frac{dB_y}{dx}x = 2\pi k_x x$ where $k_x = \frac{1}{2\pi}\gamma \frac{L}{|\bar{\mathrm{v}}|}\frac{dB_y}{dx}$. After exiting the encoding field, the direction of the magnetic moment does not change until the particle reaches the analyser. The role of the analyser is to pass the particles towards the detector with a probability which depends on ϕ and hence on the x position of the particle. Defining an ideal $\hat{\boldsymbol{x}}$-projection analyser as one with a 100% / 0% transfer probability for particles whose magnetic moment is oriented parallel or anti-parallel with respect to the +$\hat{\boldsymbol{x}}$ axis, the probability of passing into the detector as a function of ϕ can be written as $\mathrm{P}(\phi) = \frac{(\cos(\phi)+1)}{2}$. Similarly, the transition probability of passing through an ideal $\hat{\boldsymbol{z}}$-projection analyser, which is orthogonal to the original polarisation direction, can be expressed as $\mathrm{P}(\phi) = \frac{(\sin(\phi)+1)}{2}$. We will now define a complex signal, where the real part, S_0, is obtained by summing, over all N beam particles, the probability of passing through a $\hat{\boldsymbol{x}}$-projection analyser into the detector. Similarly, the imaginary part, S_{90}, sums these probabilities when passing through a $\hat{\boldsymbol{z}}$-projection analyser. Writing this explicitly we get $\mathrm{S}(k_x) = S_0 + \sqrt{-1}\, S_{90} \propto \sum_{j=1}^{N}[\cos(2\pi k_x x_j) + \sqrt{-1}\sin(2\pi k_x x_j) + 1]$ or written in compact form as $\mathrm{S}(k_x) \propto \sum_j e^{2\pi i k_x x_j} + 1$ where $i = \sqrt{-1}$. Finally, given the large number of particles in the beam, we can replace the sum with an integration within the plane perpendicular to the beam direction (xy plane). Using the density function $\rho(x,y)$ to provide the statistical weight for each position we obtain $\mathrm{S}(k_x) \propto \iint_{-\infty}^{\infty} \rho(x,y) e^{2\pi i k_x x} dxdy + C$, where the constant term C will eventually be subtracted from the measurement before the inverse Fourier transform is applied. Note that the use of infinite integration limits is justified because $\rho(x,y)$ is zero beyond the beam diameter. Rearranging the order of integration $\mathrm{S}(k_x) \propto \int_{-\infty}^{\infty}\{\int_{-\infty}^{\infty}\rho(x,y)dy\} e^{2\pi i k_x x}dx + C$ or $\mathrm{S}(k_x) \propto \int_{-\infty}^{\infty}\rho_{1\mathrm{D}}(x)\, e^{2\pi i k_x x}dx + C$, where we use $\rho_{1\mathrm{D}}(x) = \int_{-\infty}^{\infty}\rho(x,y)dy$ to denote the projection of the density distribution function $\rho(x,y)$ onto the $\hat{\boldsymbol{x}}$ axis, i.e. the 1d profile of the beam. The Fourier transform relation we obtained means that an inverse transformation $\rho_{1\mathrm{D}}(x) \propto \int_{-\infty}^{\infty}\mathrm{S}(k_x)\, e^{-2\pi i k_x x}dx$ can be used to extract the profile of the beam from the measurement of the complex signal $\mathrm{S}(k_x)$.

To extend the derivation above to 2d imaging, we can consider the effect of a second encoding element where the gradient of the field is along the y coordinate (for example $\frac{dB_y}{dy}$). This second perpendicular encoding device can be positioned either before or after the 1st encoding device, as the two encoding elements are independent, the only change in terms of the magnetic moment dynamics along the beam line, is the accumulation of an additional Larmor phase $\phi' = 2\pi k_y y$ when passing through the additional device where $k_y = \frac{1}{2\pi}\gamma\frac{L}{|\bar{\mathrm{v}}|}\frac{dB_y}{dy}$. The total phase accumulated when passing through both devices is a simple sum of the phases accumulated in each of them, and the complex signal becomes $\mathrm{S}(k_x, k_y) \propto \sum_j e^{2\pi i(k_x x_j + k_y y_j)} + C$. The important difference from the 1d case is that now each particle contributes to the signal at the detector according to its total accumulated phase, which in turn depends on its two-dimensional position x_j, y_j within the plane perpendicular to the beam propagation. From here on, the derivation is identical to the 1d case and what we get is a 2d Fourier transform relationship, $\mathrm{S}(k_x, k_y) \propto \iint \rho(x,y) e^{2\pi i[k_x x + k_y y]}dxdy + C$, between the 2d signal and the 2d density $\rho(x,y)$ which is given in Eq. 2 of the manuscript. Note that

if we eliminate the second gradient (substituting $k_y = 0$) and rearrange the integration order, we return to Eq. 1 in the main text.
