# Supplementary material for: Neutral beam microscopy with a reciprocal space approach using magnetic beam spin encoding
Source: Nat Commun. 2024 Aug 15;15:7046. doi: 10.1038/s41467-024-51175-2 (PMC11327282; doi:10.1038/s41467-024-51175-2)
Supplement: Supplementary file 3 — Description of Additional Supplementary Files [file 41467_2024_51175_MOESM3_ESM.pdf]

Description of Additional Supplementary Files

Supplementary Movie 1: Simplified animation illustrating the process of performing phase encoded imaging to a beam of atoms (represented by blue circles) moving left to right (in the $\hat{\mathbf{z}}$ direction) with a magnetic moment direction shown via the directional arrows. To start with the magnetic moments of the polarised beam are all pointing along the $\hat{\mathbf{x}}$ direction (up). The beam is partially blocked by a ‘sample’ (vertical rectangle). The beam then continues into the beam encoding region, which is identified between the two horizontal rectangular boxes. The magnetic field within the beam encoding region points along the $\hat{\mathbf{y}}$ direction (out of the screen) but changes linearly as function of the coordinate $\hat{\mathbf{x}}$, i.e. $|B| = \frac{dB_y}{dx}x$. As a result the magnetic moments of the beam particles precess with different frequencies within this region and accumulate different phases. An analyser detector combination is used to produce a signal which is the summation of the projection onto a specific axis. If we separately measure the projections along two orthogonal axes (for example along $\hat{\mathbf{x}}$ and $\hat{\mathbf{z}}$), we can store these as the real and imaginary components of a complex signal shown as blue circle and red square markers in the lower plot (the acquisition of the signal within the video assumes a simultaneous detection of both projections at once for illustrative simplicity, in practice this would be done sequentially). Note that the absence of the obstructed beam trajectories, changes the summed projections of the beam in a way which depends on the strength of the gradient. This signal acquisition process is repeated for different gradient values. Once the signal has been acquired, it is Fourier transformed and its magnitude is plotted on the right hand side, showing a spatial profile of the transmitted beam and revealing the profile of the sample which blocked the beam.

Supplementary Movie 2: Animation illustrating the ability of orthogonal fields to partially refocus the dephasing related to the velocity spread in the beam. The top panel displays a B-field profile which contains a series of 4 fields, oriented along $\hat{\mathbf{x}}$,$\hat{\mathbf{z}}$,$\hat{\mathbf{y}}$ and $\hat{\mathbf{x}}$ again representing the four fields the particles pass through in the experimental system, i.e. the dipole field of polariser, $\mathbf{B}_1$ - the solenoid field, $\mathbf{B}_2$ - the homogeneous field added to the encoding gradient field and finally the dipole field of analyser all of which were described in the main text. The

bottom right panel shows the magnetic moments of individual beam particles with different velocities as they pass through the sequence of fields, whereas the bottom left panel shows the total magnetic moment of the beam obtained by summing the moments of all the different beam particles. The evolution of the magnetic moments which are initially oriented along $\hat{\mathbf{x}}$ show the following dynamics within the 4 fields:

(0:00 – 0:04) Initial $\hat{\mathbf{x}}$ field (Polariser dipole); Since the magnetic moments are already oriented along the field direction there is no precession.

(0:08 – 0:28) $\hat{\mathbf{z}}$ field ($\mathbf{B}_1$)**;** Once entering the $\hat{\mathbf{z}}$ field, the magnetic moments start to precess within the xy plane. Particles with different velocities accumulate a different phase, and the total magnetisation (visible in the left plot) decays significantly when the phase difference exceeds 2π.

(0:32 – 0:53) $\hat{\mathbf{y}}$ field ($\mathbf{B}_2$); This second field leads to precession in the xz plane. Since the field is in the $\hat{\mathbf{y}}$ direction it can not effect the projection along this axis and can not refocus this component. The field can however refocus the spread of projections along $\hat{\mathbf{x}}$. As seen in the animation the precession leads to a regaining of coherency which can be observed by following the individual moments (bottom right panel), and more easily when looking at their sum (bottom left panel). The sum of the magnetic moments reaches a maximum value when the field integral of the two precession fields is equal. As only the $\hat{\mathbf{x}}$ component is refocused, the sum reaches only $\frac{\sqrt{2}}{2}$ its original length.

(0:57 – 1:01) final $\hat{\mathbf{x}}$ dipole field; Precession is now induced in the yz plane. An important point is the $\hat{\mathbf{x}}$ projection is not changed by this precession, as can be seen for individual moment or more easily by looking at the sum of the magnetic moments (bottom left). Since, in the experimental setup, the analyser transmits particles toward the detector in a way which depends on their $\hat{\mathbf{x}}$ projection, the signal of a measurement will not be affected by the precessions within this field.
